# Supplementary material for: Generalizable and automated classification of TNM stage from pathology reports with external validation
Source: Nat Commun. 2024 Oct 16;15:8916. doi: 10.1038/s41467-024-53190-9 (PMC11484761; doi:10.1038/s41467-024-53190-9)
Supplement: Supplementary file 1 — Supplementary Information [file 41467_2024_53190_MOESM1_ESM.pdf]

**Supplementary Material**

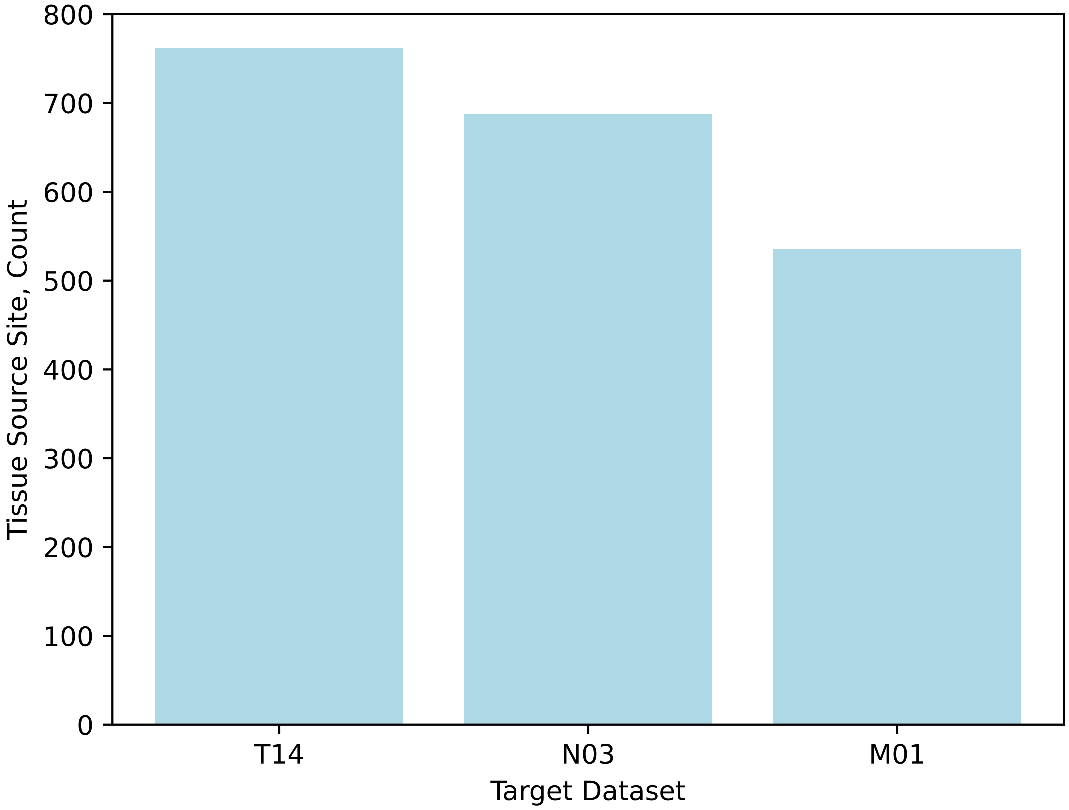


**Figure S1.** Distribution of number of tissue source sites across TCGA for each target dataset. Each tissue source site represents a different institution that contributed both slides and pathology reports to the TCGA dataset. Within each institution, there are a variety of pathologists contributing reports. As a result, each target dataset contains reports with a wide range of formatting, writing styles, and abbreviations.

**Table S1.** Tokenization statistics for TCGA training set reports. The CB Tokenizer was used. Included the number (n) and proportion (p) of reports above each threshold token limit. Model CB has a per-report 512 input token limit, whereas model CBB has an input threshold of 4,096 tokens per report.

| Maximum Tokens Threshold | Reports above Threshold (n) | Reports above Threshold (p) |
|---|---|---|
| 512 | 5345 | 66.0% |
| 1024 | 3122 | 38.6% |
| 2048 | 1045 | 12.9% |
| 4096 | 55 | 0.7% |

**Table S2.** Best-performing model parameters, by classification target, based on TCGA validation set performance. Included parameters are model type, batch size, and maximum tokens per input report. Batch size was limited by memory constraints. ClinicalBERT was not found to be best-performing for any of the target classification tasks.

| Target | Model Type | Batch Size | Tokens/Input |
|---|---|---|---|
| **T14** | Clinical-BigBird | 2 | 2048 |
| **N03** | Clinical-BigBird | 2 | 2048 |
| **M01** | Clinical-BigBird | 4 | 1024 |

A)

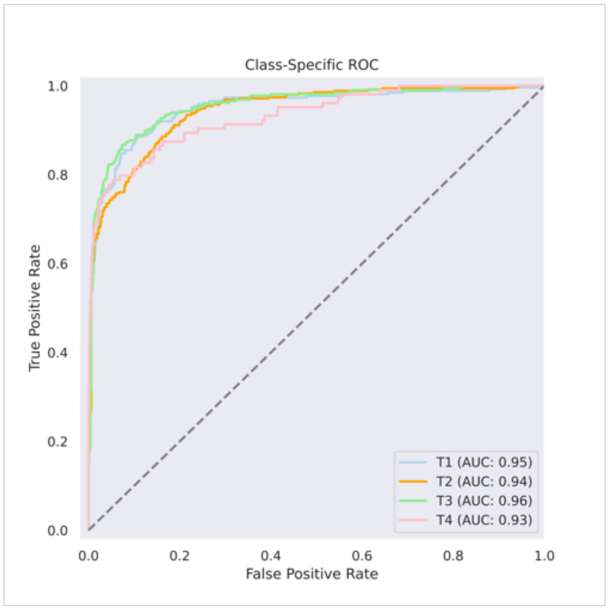

B)

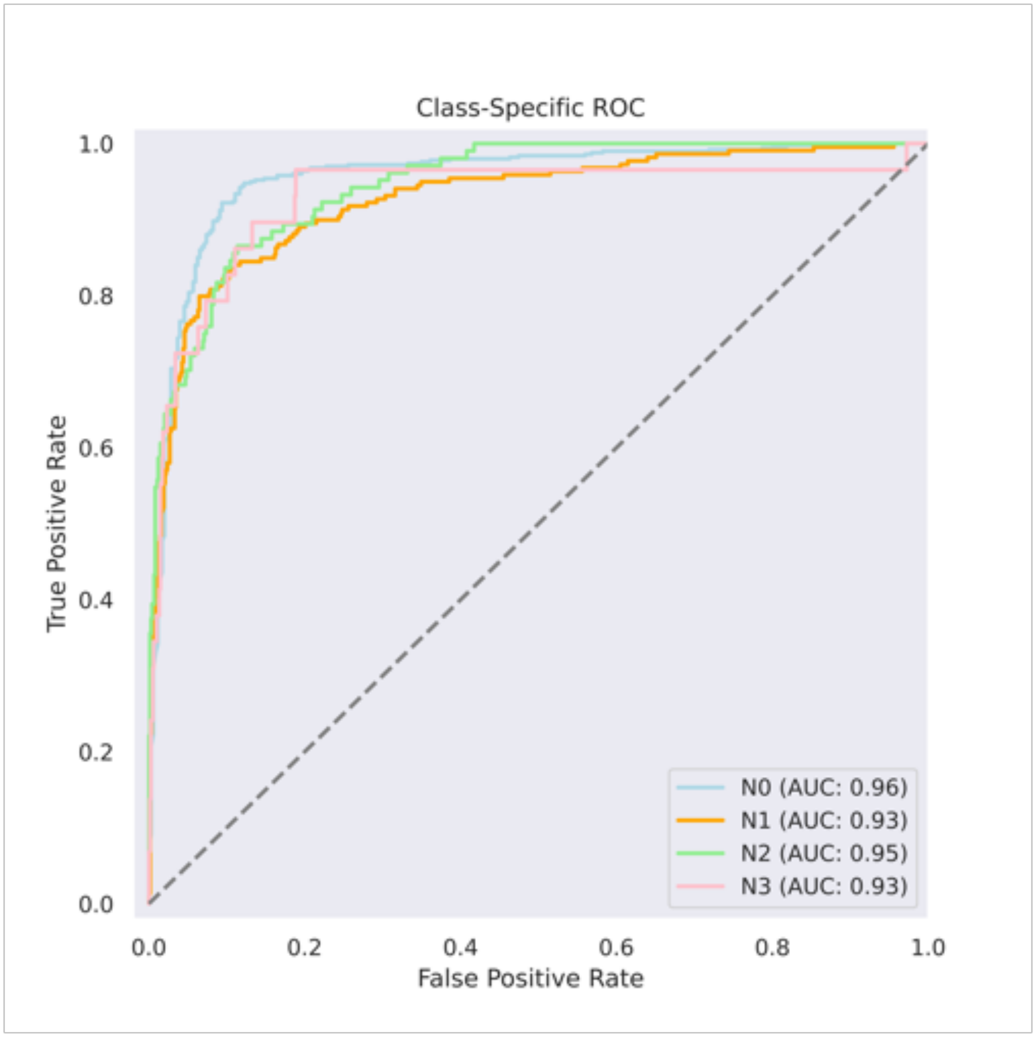

Class-Specific ROC
True Positive Rate
False Positive Rate
0.0
0.2
0.4
0.6
0.8
1.0
N0 (AUC: 0.96)
N1 (AUC: 0.93)
N2 (AUC: 0.95)
N3 (AUC: 0.93)

C)

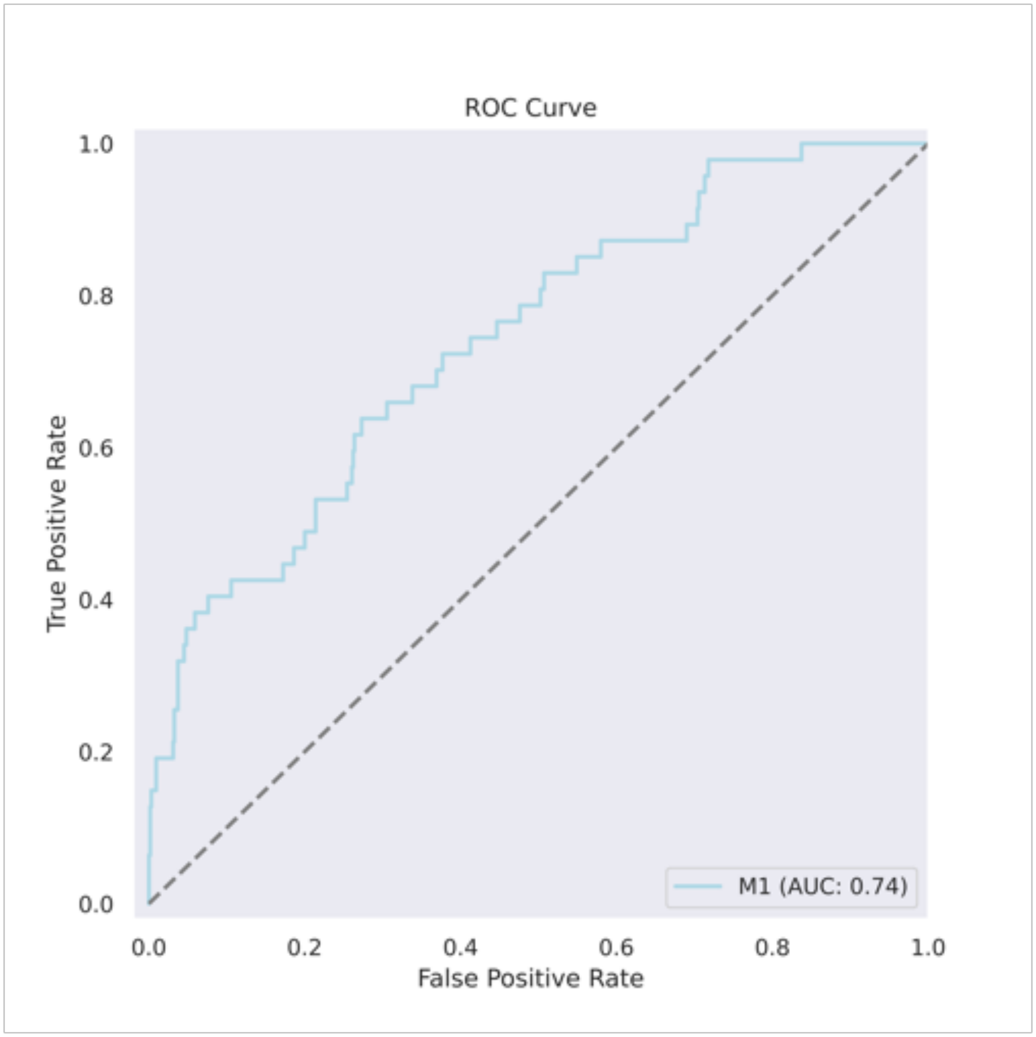

ROC Curve
True Positive Rate
False Positive Rate
0.0
0.2
0.4
0.6
0.8
1.0
M1 (AUC: 0.74)

**Figure S2.** Overall model performance on held-out TCGA test set. ROC curves for (A) T14, (B) N03, and (C) M01 models. Individual AU-ROC is captured in each plot legend. For (A) and (B), each curve corresponds to per-class performance, calculated as one-versus-all.

(A)
(i)

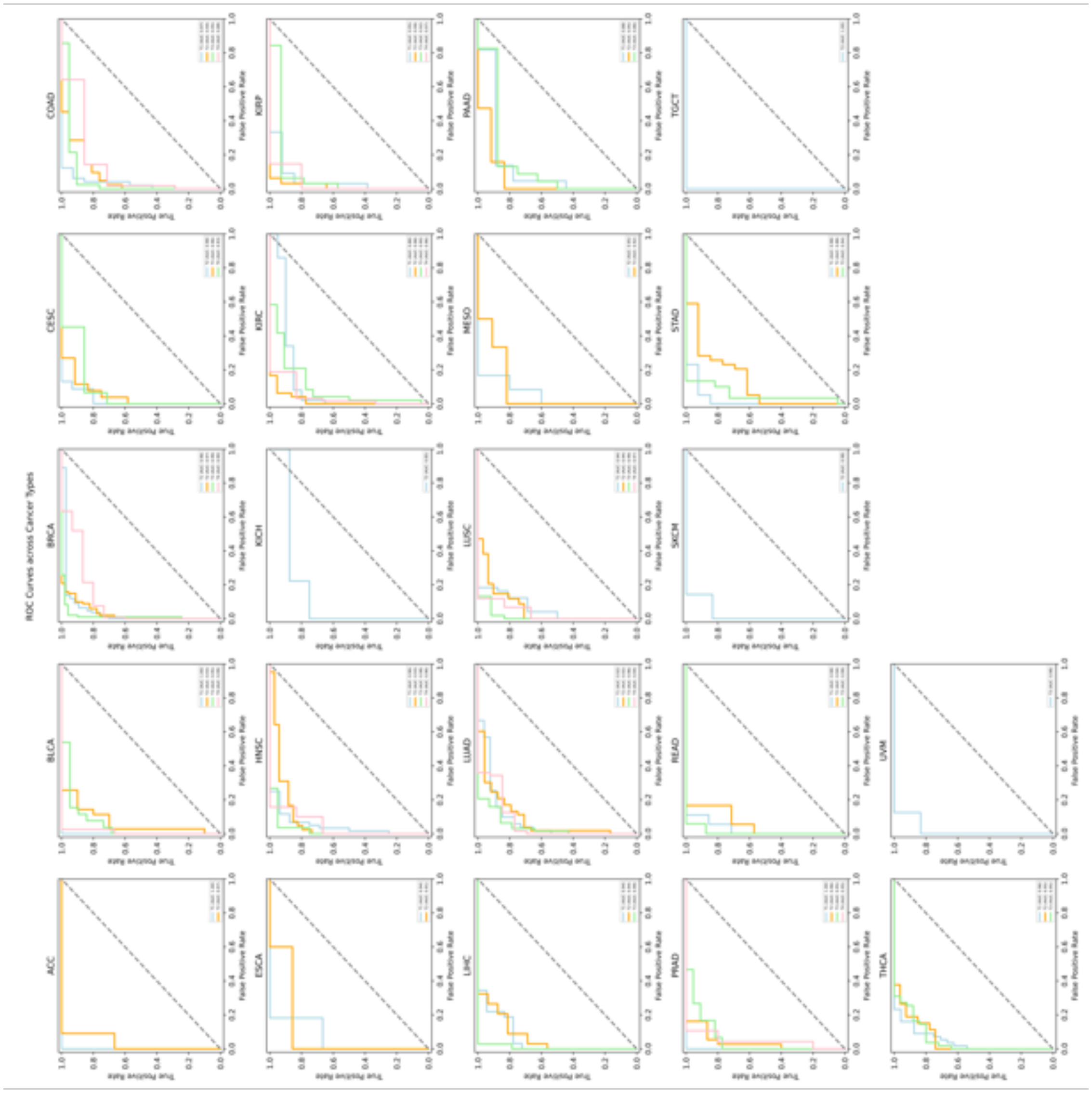

(ii)

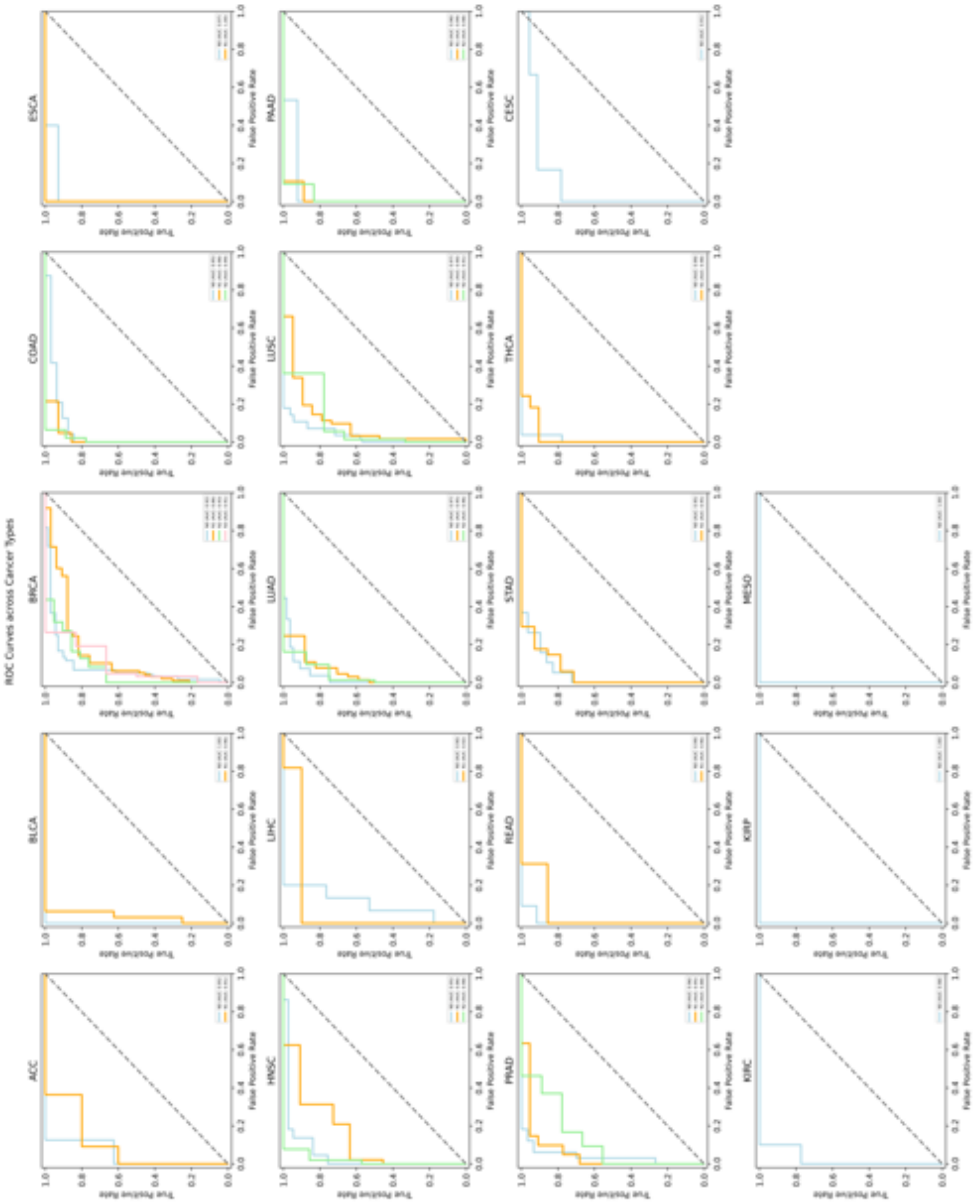

ROC Curves across Cancer Types
ACC
BLCA
BRCA
COAD
ESCA
HNSC
LIHC
LUAD
LUSC
PAAD
PRAD
READ
STAD
THCA
CESC
KIRC
KIRP
MESO
False Positive Rate
True Positive Rate

(B)
(i)

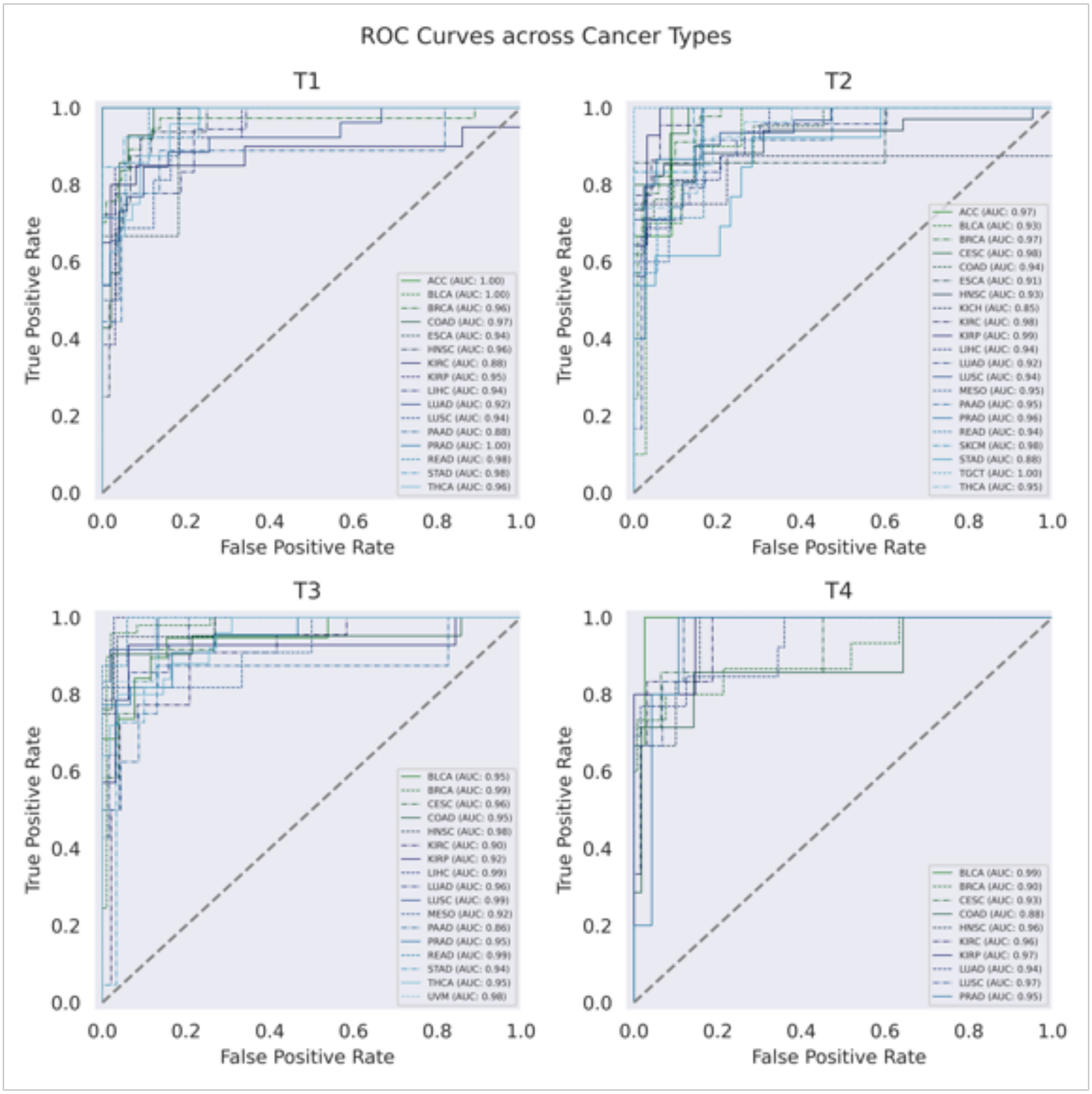
ROC Curves across Cancer Types
T1
True Positive Rate
False Positive Rate
ACC (AUC: 1.00)
BLCA (AUC: 1.00)
BRCA (AUC: 0.96)
COAD (AUC: 0.97)
ESCA (AUC: 0.94)
HNSC (AUC: 0.96)
KIRC (AUC: 0.88)
KIRP (AUC: 0.95)
LIHC (AUC: 0.94)
LUAD (AUC: 0.92)
LUSC (AUC: 0.94)
PAAD (AUC: 0.88)
PRAD (AUC: 1.00)
READ (AUC: 0.98)
STAD (AUC: 0.98)
THCA (AUC: 0.96)
T2
True Positive Rate
False Positive Rate
ACC (AUC: 0.97)
BLCA (AUC: 0.93)
BRCA (AUC: 0.97)
CESC (AUC: 0.98)
COAD (AUC: 0.94)
ESCA (AUC: 0.91)
HNSC (AUC: 0.93)
KICH (AUC: 0.85)
KIRC (AUC: 0.98)
KIRP (AUC: 0.99)
LIHC (AUC: 0.94)
LUAD (AUC: 0.92)
LUSC (AUC: 0.94)
MESO (AUC: 0.95)
PAAD (AUC: 0.95)
PRAD (AUC: 0.96)
READ (AUC: 0.94)
SKCM (AUC: 0.98)
STAD (AUC: 0.88)
TGCT (AUC: 1.00)
THCA (AUC: 0.95)
T3
True Positive Rate
False Positive Rate
BLCA (AUC: 0.95)
BRCA (AUC: 0.99)
CESC (AUC: 0.96)
COAD (AUC: 0.95)
HNSC (AUC: 0.98)
KIRC (AUC: 0.90)
KIRP (AUC: 0.92)
LIHC (AUC: 0.99)
LUAD (AUC: 0.96)
LUSC (AUC: 0.99)
MESO (AUC: 0.92)
PAAD (AUC: 0.86)
PRAD (AUC: 0.95)
READ (AUC: 0.99)
STAD (AUC: 0.94)
THCA (AUC: 0.95)
UVM (AUC: 0.98)
T4
True Positive Rate
False Positive Rate
BLCA (AUC: 0.99)
BRCA (AUC: 0.90)
CESC (AUC: 0.93)
COAD (AUC: 0.88)
HNSC (AUC: 0.96)
KIRC (AUC: 0.96)
KIRP (AUC: 0.97)
LUAD (AUC: 0.94)
LUSC (AUC: 0.97)
PRAD (AUC: 0.95)

(ii)

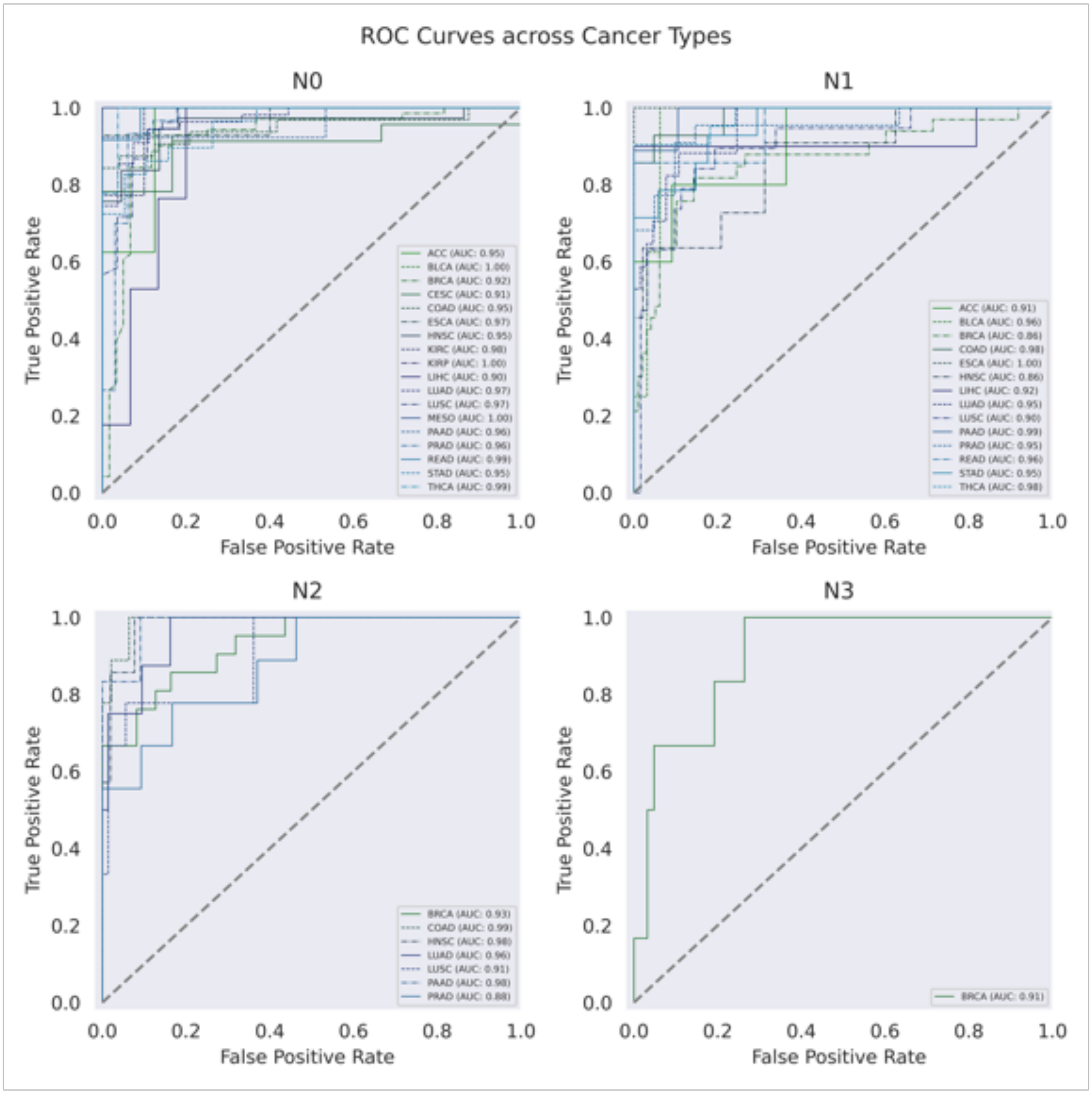
ROC Curves across Cancer Types
N0
True Positive Rate
False Positive Rate
ACC (AUC: 0.95)
BLCA (AUC: 1.00)
BRCA (AUC: 0.92)
CESC (AUC: 0.91)
COAD (AUC: 0.95)
ESCA (AUC: 0.97)
HNSC (AUC: 0.95)
KIRC (AUC: 0.98)
KIRP (AUC: 1.00)
LIHC (AUC: 0.90)
LUAD (AUC: 0.97)
LUSC (AUC: 0.97)
MESO (AUC: 1.00)
PAAD (AUC: 0.96)
PRAD (AUC: 0.96)
READ (AUC: 0.99)
STAD (AUC: 0.95)
THCA (AUC: 0.99)
N1
True Positive Rate
False Positive Rate
ACC (AUC: 0.91)
BLCA (AUC: 0.96)
BRCA (AUC: 0.86)
COAD (AUC: 0.98)
ESCA (AUC: 1.00)
HNSC (AUC: 0.86)
LIHC (AUC: 0.92)
LUAD (AUC: 0.95)
LUSC (AUC: 0.90)
PAAD (AUC: 0.99)
PRAD (AUC: 0.95)
READ (AUC: 0.96)
STAD (AUC: 0.95)
THCA (AUC: 0.98)
N2
True Positive Rate
False Positive Rate
BRCA (AUC: 0.93)
COAD (AUC: 0.99)
HNSC (AUC: 0.98)
LUAD (AUC: 0.96)
LUSC (AUC: 0.91)
PAAD (AUC: 0.98)
PRAD (AUC: 0.88)
N3
True Positive Rate
False Positive Rate
BRCA (AUC: 0.91)

(iii)

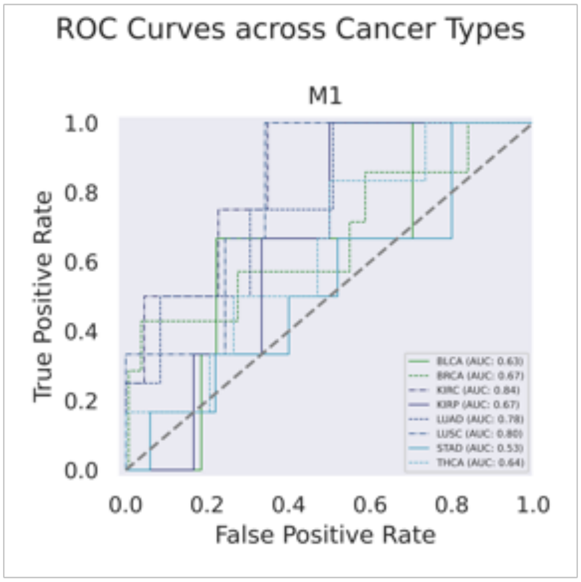


**Figure S3.** Per-class and per-cancer type best-model performance on held-out TCGA test set. (A) Per-cancer type, across classes. (i-ii) T14, N03. (B) Per-class, across cancer types. (i-iii) T14, N03, M01. At least 5 examples per class in the test set were required for cancer type inclusion,

except for M01, for which the threshold was reduced to 3 test examples per class due to low-data availability. AU-ROC is presented in each plot legend.

**Table S3.** CUIMC report set characterization. (A) Compiled CUIMC report statistics, number of patients and primary cancer sites. (B) Compiled CUIMC report demographic statistics, including sex, age, self-reported race and self-reported ethnicity. The missing percentages represent other or unknown. (C) Tokens per input for CUIMC pathology reports, using CBB Tokenizer. (D) CUIMC class distribution, by target (T, N, M). (E) Performance of TNM models on CUIMC data, as compared to TNM model performance in (Abedian et al., 2021), across all cancer types. For (Abedian et al., 2021) results, we selected the pan-cancer "random subtypes" test set; this matched most closely with the inclusion of all subtypes in the CUIMC dataset. F1 is micro-computed.

**(A)**

| Target | Patients (n) | Primary Sites (n) |
|---|---|---|
| **T14** | 7,792 | 42 |
| **N03** | 6,140 | 41 |
| **M01** | 2,245 | 40 |

**(B)**

| Target | T14 | N03 | M01 |
|---|---|---|---|
| **Female (%)** | 57.25 | 56.01 | 58.44 |
| **Average age (years)** | 67.07 | 67.73 | 65.99 |
| **STD age (years)** | 15.22 | 15.07 | 15.74 |
| **Race: Asian (%)** | 3.29 | 3.25 | 3.35 |
| **Race: Black (%)** | 8.93 | 9.12 | 9.17 |
| **Race: White (%)** | 54.64 | 52.83 | 52.23 |
| **Ethnicity: Hispanic (%)** | 16.56 | 17.09 | 18.25 |
| **Ethnicity: Not Hispanic (%)** | 54.57 | 52.72 | 54.34 |

**(C)**

| Target | Max Tokens | Average Tokens | Median Tokens |
|---|---|---|---|
| **T14** | 9104 | 1915.1 | 1683 |
| **N03** | 9104 | 2092.1 | 1848 |
| **M01** | 9776 | 1963.5 | 1730 |

(D)

| Target | T1 | T2 | T3 | T4 |
|---|---|---|---|---|
| **T14** | 45.8% | 24.2% | 25.4% | 4.5% |

| **Target** | N0 | N1 | N2 | N3 |
|---|---|---|---|---|
| **N03** | 69.7% | 24.0% | 5.1% | 1.2% |

| **Target** | M0 | M1 |
|---|---|---|
| **M01** | 79.9% | 20.1% |

(E)

| **Target** | **F1** (Kefeli, 2023) | **F1** (Abedian, 2021) |
|---|---|---|
| **T14** | 0.78 | 0.78 |
| **N03** | 0.86 | 0.78 |
| **M01** | 0.82 | 0.11 |

(A)

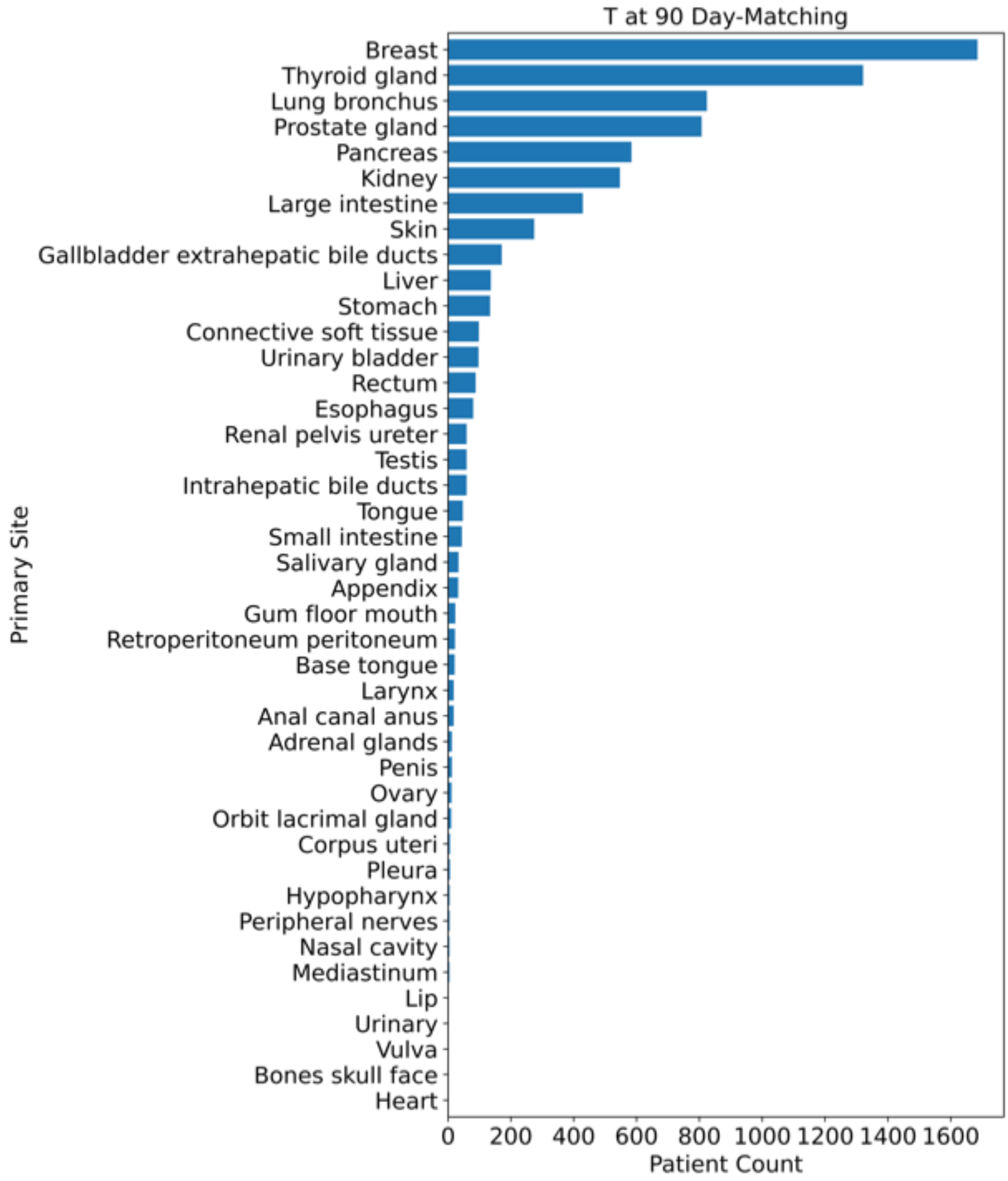
T at 90 Day-Matching
Primary Site
Breast
Thyroid gland
Lung bronchus
Prostate gland
Pancreas
Kidney
Large intestine
Skin
Gallbladder extrahepatic bile ducts
Liver
Stomach
Connective soft tissue
Urinary bladder
Rectum
Esophagus
Renal pelvis ureter
Testis
Intrahepatic bile ducts
Tongue
Small intestine
Salivary gland
Appendix
Gum floor mouth
Retroperitoneum peritoneum
Base tongue
Larynx
Anal canal anus
Adrenal glands
Penis
Ovary
Orbit lacrimal gland
Corpus uteri
Pleura
Hypopharynx
Peripheral nerves
Nasal cavity
Mediastinum
Lip
Urinary
Vulva
Bones skull face
Heart
0 200 400 600 800 1000 1200 1400 1600
Patient Count

(B)

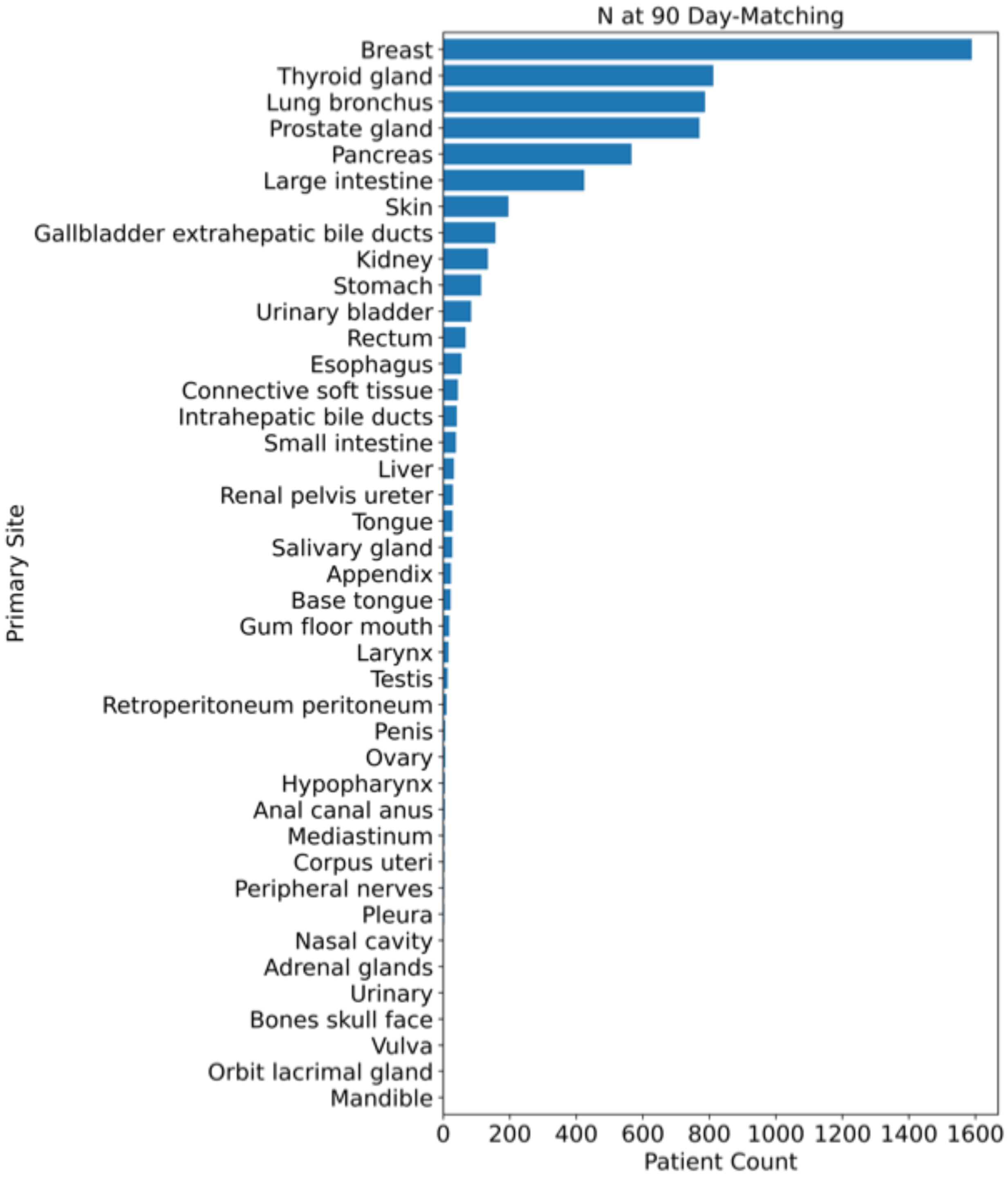

N at 90 Day-Matching
Breast
Thyroid gland
Lung bronchus
Prostate gland
Pancreas
Large intestine
Skin
Gallbladder extrahepatic bile ducts
Kidney
Stomach
Urinary bladder
Rectum
Esophagus
Connective soft tissue
Intrahepatic bile ducts
Small intestine
Liver
Renal pelvis ureter
Tongue
Salivary gland
Appendix
Base tongue
Gum floor mouth
Larynx
Testis
Retroperitoneum peritoneum
Penis
Ovary
Hypopharynx
Anal canal anus
Mediastinum
Corpus uteri
Peripheral nerves
Pleura
Nasal cavity
Adrenal glands
Urinary
Bones skull face
Vulva
Orbit lacrimal gland
Mandible
Primary Site
0
200
400
600
800
1000
1200
1400
1600
Patient Count

(C)

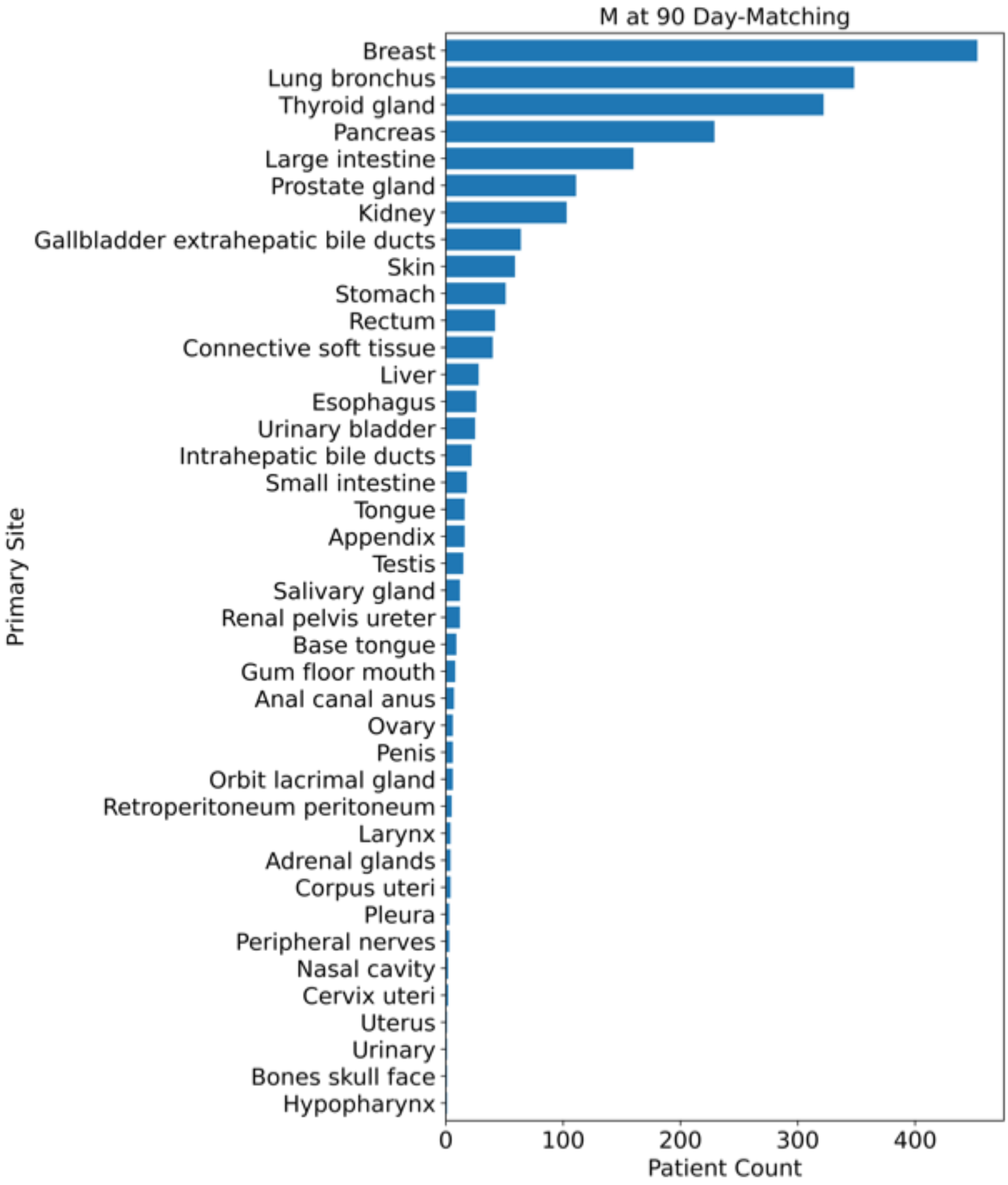


Figure S4. Primary site distribution of CUIMC reports. (A) T14, (B) N03, (C) M01.

**Table S4.** CUIMC Performance on N03 target with TCGA-trained ClinicalBERT, 512 input tokens (CB-512), and TCGA-trained Clinical-BigBird, 2,048 input tokens (CBB-2048). AU-ROC macro-computed.

| Model | AU-ROC | F1 Micro | AU-ROC (N0) | AU-ROC (N1) | AU-ROC (N2) | AU-ROC (N3) |
|---|---|---|---|---|---|---|
| CB-512 | 0.779 | 0.688 | 0.782 | 0.782 | 0.764 | 0.789 |
| CBB-2048 | 0.912 | 0.863 | 0.937 | 0.922 | 0.916 | 0.872 |

**Table S5.** CUIMC Performance on T14 target, with and without PHI preamble per report. AU-ROC macro-computed.

| Condition | AU-ROC | F1 Micro |
|---|---|---|
| With PHI | 0.9415 | 0.7767 |
| Without PHI | 0.9416 | 0.7862 |

(A)

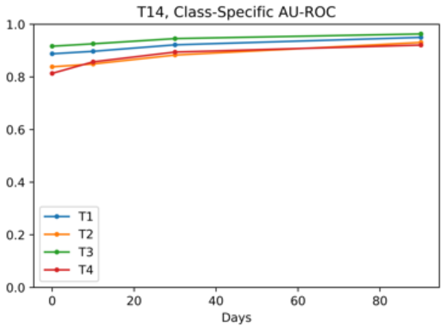

(B)

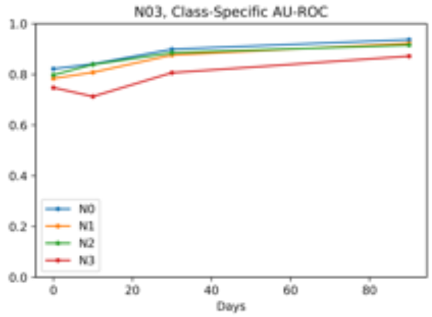
N03, Class-Specific AU-ROC
1.0
0.8
0.6
0.4
0.2
0.0
0
20
40
60
80
Days
N0
N1
N2
N3

(C)

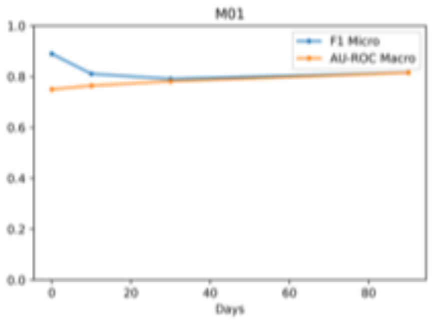


Figure S5. Sensitivity analysis, AU-ROC values with increasing number of days (time-windows) between pathology report and documented diagnosis (for report-matching process, see Methods).

(A) T14, (B) N03, (C) M01. For (A) and (B), AU-ROC is computed per-class for (C), which is binary, AU-ROC and F1-micro are plotted.

Table S6. Computation time for training models.

| Model | All params (millions) | Trainable params (millions) | Training time |
|---|---|---|---|
| ClinicalBERT | 108.3 | 108.3 | 0.05h/epoch |
| Clinical_Bigbird | 128.1 | 128.1 | 0.12h/epoch |
| Llama3-FT | 8118.4 | 88.1 | 1.08h/epoch |
